# Tumor necrosis is a strong predictor for recurrence in patients with pathological T1a renal cell carcinoma

**DOI:** 10.3892/ol.2014.2670

**Published:** 2014-11-04

**Authors:** KEIICHI ITO, KENJI SEGUCHI, HIDEYUKI SHIMAZAKI, EIJI TAKAHASHI, SHINSUKE TASAKI, KENJI KURODA, AKINORI SATO, JUNICHI ASAKUMA, AKIO HORIGUCHI, TOMOHIKO ASANO

**Affiliations:** 1 Department of Urology, National Defense Medical College, Tokorozawa, Saitama 359-8513, Japan; 2 Department of Laboratory Medicine, National Defense Medical College, Tokorozawa, Saitama 359-8513, Japan

**Keywords:** renal cell carcinoma, pathological T1a, recurrence, predictor, tumor necrosis

## Abstract

Patients with pT1aN0M0 renal cell carcinoma (RCC) generally have good prognosis, and recurrence is rare. However, metastasis develops postoperatively in a small number of patients with pT1aN0M0 RCC. The present study was undertaken to identify predictors for recurrence in patients with pT1aN0M0 RCC. We reviewed the clinicopathological factors of 133 patients with pT1aN0M0 RCC who underwent radical or partial nephrectomy at the Department of Urology, National Defense Medical College (Saitama, Japan). Clinicopathological factors, including age, gender, tumor size, histological subtype, tumor grade, microvascular invasion, histological tumor necrosis, C-reactive protein levels and performance status were reviewed. These factors were compared between patients with and without postoperative recurrence. Recurrence-free survival (RFS) and cause-specific survival (CSS) rates were calculated using the Kaplan-Meier method. Univariate and multivariate analyses were performed to determine independent factors predicting recurrence in patients with pT1aN0M0 RCC. The 5-year RFS and CSS rates were 97.2 and 99.1%, respectively. When clinicopathological factors were compared between patients with and without recurrence, tumor size (P=0.0390) and percentage of tumor necrosis (P<0.0001) were significantly different between groups. All patients with recurrence had primary lesions ≥3 cm. By univariate analysis, tumor size (P=0.0379) and the presence of tumor necrosis (P=0.0319) were significant predictors for recurrence; tumor necrosis was also an independent predictor for recurrence (P=0.0143). In patients with pT1b tumors ≤5 cm (recurrence rate, 16.8%; n=48), the percentage of tumor necrosis was significantly higher in patients with recurrence compared with those without (P=0.0261). This suggests that tumor necrosis may be an important predictor for recurrence in small RCCs. Although recurrence is rare in pT1a RCC, the presence of tumor necrosis may be an important predictor for recurrence. Particularly, patients presenting with pT1a RCC with histological tumor necrosis should undergo careful follow-up.

## Introduction

The prognosis of patients with T1aN0M0 renal cell carcinoma (RCC) is favorable, and recurrence is rare. Risk factors for recurrence in clinical T1a (cT1a) RCC have been previously evaluated (1–4). Takayama *et al* reported that symptomatic cancer, sarcomatoid component, and C-reactive protein (CRP) levels ≥0.4 mg/dl were risk factors for recurrence in cT1a RCC (1). In addition, Kume *et al* reported that microvascular invasion (MVI) was an independent predictor for distant metastasis of RCC with a diameter of ≤3 cm (2). Since patients with cT1a RCC include those with pathological T3a (pT3a) RCC, cT1a tumors theoretically, frequently include more aggressive tumors compared with patients with pT1a tumors. Although pT1a RCC tumors generally recur less frequently than cT1a, there are a small number of patients with pT1a disease recurrence.

Two studies have evaluated the predictors for recurrence in patients with pT1a RCC (5,6). Kim *et al* (5) revealed that MVI and tumor necrosis were independent predictors for recurrence. Nishikimi *et al* (6) evaluated RCC patients with clear cell RCC using multivariate analysis and found that Fuhrman grade, growth pattern and tumor necrosis were significantly associated with disease-free survival. As the majority of pT1a RCCs are less aggressive and recurrence is rare, longer follow-up intervals are generally accepted compared with RCCs at higher pathological stages (7). Kim *et al* (5) reported that 9 out of 93 pT1aN0M0 patients exhibited distant metastasis (mean follow-up duration, 63.6 months). Furthermore, Nishikimi *et al* (6) reported that 25 of 293 pT1aN0M0 patients exhibited distant metastasis (median follow-up duration, 62 months). If patients with pT1a RCC with a high risk of recurrence are identified, clinicians can monitor these patients closely and counsel them regarding the risk for recurrence.

The aim of the current study was to identify the risk factors for predicting recurrence in patients with pT1aN0M0 RCC. We evaluated the clinical characteristics of patients with pT1aN0M0 RCC in whom the disease recurred. In addition, we assessed the clinical characteristics of patients with pT1bN0M0 RCC ≤5 cm, who had a recurrence.

## Patients and methods

We reviewed the medical records of patients with RCC undergoing radical nephrectomy (RN) or partial nephrectomy (PN) at the Department of Urology, National Defense Medical College (Saitama, Japan) between 1990 and 2011. The study cohort consisted of 133 patients in whom neither preoperative radiological or pathological examination of surgical specimens indicated distant or lymph node metastasis (N0M0 patients), and whose tumors were pathologically confirmed as pT1a. Of these patients, 101 underwent RN and 32 underwent PN. Their ages ranged from 32 to 89 years (mean, 60.8±12.2). Local recurrence and metastasis were monitored by examining each patient postoperatively at 3–6 month intervals for the first 5 years, and every 6–12 months thereafter. Follow-up included physical examination, laboratory tests, chest radiography, abdominal and chest computed tomography and, if necessary, radionuclide bone scanning. The total follow-up time ranged from 1 to 261 months (median, 57.8). Recurrence-free survival (RFS) was evaluated using the date at which local recurrence or metastatic disease was identified, and overall survival (OS) was determined using either the date of death or the date of the last follow-up examination.

The clinicopathological factors evaluated are listed in Table I, and included age, gender, tumor size, histological subtype, histological tumor grade, MVI, histological tumor necrosis, CRP levels, and Eastern Cooperative Oncology Group performance status (ECOG PS) (8). These factors were compared between patients with recurrence postoperatively (n=5, 3.8%), and those without (n=128). Tumors were staged according to the 2002 TNM classification system (9), and nucleolar grading in a three-grade system was determined (10). Tumor necrosis was defined as microscopic coagulative necrosis (6,11); the presence of necrosis that was apparent on gross examination was excluded. Preoperative elevation of CRP was defined as CRP ≥0.3 mg/dl, as previously described (12,13).

We also reviewed the clinicopathological factors of patients with pT1b tumors ≤5 cm, and of those with and without recurrence.

### Statistical analysis

Results are presented as the mean ± standard deviation, and differences in variables between groups were compared using the Mann-Whitney U test. The independence of fit of categorical data was analyzed using the χ^2^ test. Survival curves were constructed using the Kaplan-Meier method, and differences between groups were assessed using the log-rank test. To determine independent factors predicting recurrence in patients with pT1aN0M0 RCC, univariate and multivariate analyses were performed using the Cox proportional-hazards regression model. P<0.05 was considered to indicate a statistically significant difference.

## Results

### Clinicopathological characteristics of patients with disease recurrence (Table I)

Five out of 133 patients with pT1a (3.8%) exhibited disease recurrence (median follow-up, 57.8 months). The mean age of these five patients (three males and two females) was 60.8 years (56–75). Four patients underwent right nephrectomy and one underwent left nephrectomy. The mean diameter of the five tumors was 3.5 cm, and all were ≥3 cm. The ECOG PS in three patients was 0, in one patient was 1 and in the remaining patient was 3. Metastases were detected in the lungs of three patients, the mediastinal lymph node in one and the contralateral kidney of one patient. The time from nephrectomy to recurrence was <1 year in two patients (3.5 and 5.9 months), and >4 years in three patients (48.2, 61.2 and 77.5 months). All five patients had clear cell-type RCC; four tumors were histological grade 2 and one was grade 3. Two of the five tumors (40%) had microvascular invasion and three (60%) had histological tumor necrosis. Two patients (40%) had preoperative CRP levels ≥0.3 mg/dl. No patients had thrombocytosis.

### Comparison of clinicopathological factors between patients with and without recurrence (Table I)

Tumor size and the percentage of tumor necrosis were significantly higher in patients with recurrence than in those without. Age (P=0.2636), gender (P=0.5930), size of the tumor (P=0.1769), ECOG PS (P=0.0778), RCC subtype (P=0.6203), the presence of grade 3 component (P=0.4325), the presence of MVI (P=0.1114) and CRP (P=0.0515) were not significantly different between the two groups.

### Impact of clinicopathological factors on recurrence in patients with pT1aN0M0 RCC

**Figure 1:**
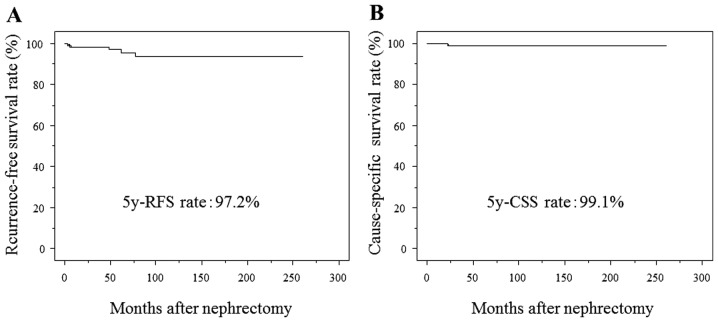
Kaplan-Meier curves analyzing recurrence-free survival (RFS) and cause-specific survival (CSS) in patients with pT1aN0M0 RCC. The 5-year (A) RFS and (B) CSS rates were 97.2 and 99.1%, respectively.

**Figure 2:**
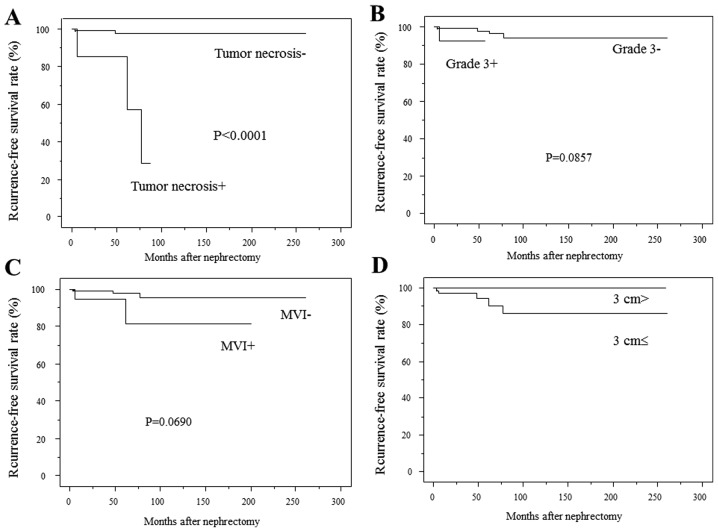
Kaplan-Meier curves analyzing recurrence-free survival (RFS). (A) Recurrence was significantly higher in patients with histological tumor necrosis than in those without. The 5- and 7-year RFS rates were 85.7 and 28.6% in patients with tumor necrosis, and were 97.9 and 97.9% in patients without tumor necrosis, respectively. (B) and (C) Recurrence rates were comparable between patients with and without a grade 3 component (B), and between patients with and without microvascular invasion (MVI) (C). (D) Patients with tumors <3 cm had no recurrence in the present study.

In all patients with pT1aN0M0 RCC, the 5-year RFS and CSS rates were 97.2 and 99.1%, respectively (Fig. 1). Kaplan-Meier analysis revealed that the recurrence rate was significantly higher in patients with histological tumor necrosis than in those without (P<0.0001) (Fig. 2A). The 5- and 7-year RFS rates were 85.7 and 28.6% in patients with tumor necrosis, and 97.9 and 97.9% in patients without tumor necrosis, respectively. The recurrence rates were not significantly different between patients with a grade 3 component and those without (Fig. 2B), or between patients with and without MVI (Fig. 2C). Patients with a tumor size <3 cm had no recurrence (Fig. 2D).

### Factors predicting recurrence in patients with pT1aN0M0 RCC

The Cox proportional-hazards regression model was used to evaluate factors predicting recurrence. Univariate analysis showed that tumor size (P=0.0379) and presence of tumor necrosis (P=0.0003) were significantly associated with RFS. Multivariate Cox proportional-hazards regression model analysis revealed that the presence of tumor necrosis was the only significant predictor of RFS (P=0.0143) (Table II).

### Comparison of clinicopathological factors in patients with pT1b ≤5 cm with and without recurrence

**Figure 3:**
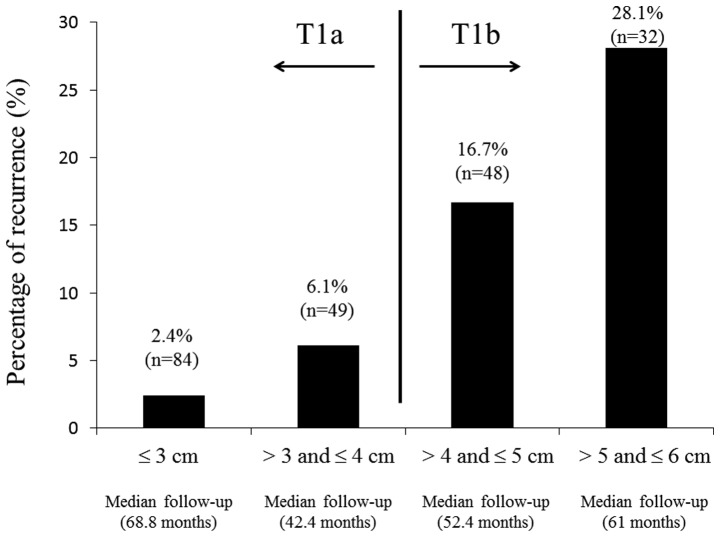
The percentage of renal cell carcinoma patients with different sized tumors. The percentage of patients with pT1aN0M0 >3 cm (n=49, median follow-up 42.4 months) was 6.1%, where as 16.7% of patients had pT1b ≤5 cm (n=48, median follow-up 52.4 months).

We reviewed the clinicopathological factors of patients with tumors larger than pT1a tumors (pT1b tumors >4cm-≤5 cm) with and without recurrence. As shown in Fig. 3, the percentage of RCC patients with recurrence gradually increased according to tumor size. The percentage of patients with pT1aN0M0 >3 cm (n=49; median follow-up time, 42.4 months) was 6.1%, whereas the percentage of patients with pT1b ≤5 cm (n=48; median follow-up time, 52.4 months) was 16.7%. When the clinicopathological factors of patients with pT1b tumors ≤5 cm with and without recurrence were compared, the percentage of tumor necrosis (P=0.0261) and gender (P=0.0367) were significantly different (Table III), suggesting that tumor necrosis may be an important predictor for the recurrence of small RCCs.

## Discussion

In the present study, five of 133 patients with pT1aN0M0 RCC (3.8%) experienced tumor recurrence (median follow-up time, 57.8 months). In previous studies, the 5-year RFS rates were 88–93% in patients with pT1aN0M0 RCC (5,6). The 5-year RFS rate in our study was higher than that in the previous studies. In the current study, patients with recurrence had a significantly increased tumor size and a higher percentage of tumor necrosis compared with patients without recurrence. Univariate analysis for the prediction of recurrence revealed that tumor size and necrosis were significant factors, but only tumor necrosis was an independent predictor for recurrence using multivariate analysis. When patients with pT1bN0M0 RCC with tumors sized ≤5 cm were evaluated, the percentage of tumor necrosis was higher in patients with recurrence compared with without recurrence. Therefore, tumor necrosis appeared to be a strong predictor for recurrence in small RCCs.

Predictors for recurrence and prognosis in cT1a RCC have been previously evaluated (1–4). Takayama *et al* (1) reported that symptomatic cancer and the presence of sarcomatoid components were independent risk factors for metachronous metastasis, and CRP levels of ≥0.4 mg/dl were an independent prognostic factor for overall survival. Kume *et al* (2) reported that MVI was an independent predictor for metastasis (2). Furthermore, cT1a RCC patients with tumors ≥3.1 cm exhibited lower recurrence-free survival rates than those with tumor ≤3.0 cm, and patients with MVI exhibited lower recurrence-free survival rates than those without MVI (3). By contrast, tumor size not identified as an independent progostic factor in RCC patients with tumors ≤4 cm (4). We hypothesize that tumors in patients with cT1a RCC are theoretically more aggressive than those with pT1a RCC. In previous studies, the common site of recurrence in patients with cT1a RCC was the bone (1,2). Takayama *et al* reported that 65% of patients with cT1a with simultaneous or metachronus metastasis had bone metastasis (1). The authors also reported that the presence of a sarcomatoid component was an independent predictor for prognosis, and four out of the five patients with a sarcomatoid component exhibited bone metastases. Consistent with this, Nishikimi *et al* reported that the bone was a predominant site of recurrence (10 of 25 recurrent patients, 40%) in patients with pT1aN0M0 RCC (6). However, the mechanism for the preference of bone metastasis in cT1a RCC remains unclear. By contrast, there were no patients with bone recurrence in the present study. Kim *et al* reported that the lungs were the major site of recurrence in patients with pT1aN0M0RCC (four of nine patients), and only one patient had a recurrence in the bone (5). Therefore, it remains controversial whether the bone is a preferred site of recurrence in pT1aN0M0 RCC.

A small number of studies have set out to identify predictors for recurrence in pT1aN0M RCC. Nishikimi *et al* reported that Fuhrman nucleolar grade, growth pattern and tumor necrosis were independent predictors for recurrence in pT1aN0M0 clear cell RCC (6). In addition, Kim *et al* reported that microvascular invasion and tumor necrosis were independent predictors for distant metastasis in pT1aN0M0 RCC (5). Consistent with these two studies, the current study identified that tumor necrosis was an independent predictor for recurrence, suggesting that tumor necrosis may be an important predictor for the recurrence of pT1aN0M0 RCC.

In the present study, we also evaluated pT1bN0M0 RCC with a tumor size ≤5 cm. In this population with relatively small pT1b tumors, patients with a recurrence had a significantly higher percentage of tumor necrosis than those without recurrence (50 vs. 15%, P=0.0261). This suggests that tumor necrosis may predict the recurrence of small RCCs, which generally have low recurrence rates. Moreover, we have previously demonstrated that the non-normalization of postoperative CRP, pre-CRP elevation, microvascular invasion, and histological tumor necrosis were independent predictors for recurrence in N0M0 clear cell RCC (13). Therefore, tumor necrosis appears to accurately reflect biological activity, tumor grade and microvascular invasion; thus, it may predict recurrence of RCC.

MVI is an important predictor for recurrence in low clinical stage RCC (2,3,14). In our five patients with recurrence, two (40%) had MVI. In addition, four of the five patients with recurrence had distant visceral metastases, and one had LN metastasis. Distant and LN metastasis theoretically require MVI. Therefore, tumors with a small degree of MVI may be occasionally diagnosed as lacking MVI. In contrast, tumor necrosis usually occupies a relatively large area in RCC specimens compared with MVI. Therefore, the presence of tumor necrosis is unlikely to be missed during pathological diagnosis.

Identifying the risk factors for recurrence may be useful for determining the optimal follow-up period in patients with pT1a RCC. Antonelli *et al* defined a follow-up protocol based on the University of California Los Angelus Integrated Staging System (15) after surgery for N0M0 RCC (16). In their study, pT1 low-risk patients (pT1 and nucleolar grade 1–2, ECOG PS=0) required thoracic examination every 30 months and abdominal examination annually for 5 years after surgery. In addition, Hafez reported that annual follow-up with a medical history, physical examination, and select laboratory studies were sufficient for patients with RCC ≤2.5 cm (17). If we can establish a risk classification system that includes tumor necrosis as a predictor for recurrence, it may be possible to more effectively predict recurrence in patients with pT1a RCC. Therefore, risk classification may be useful for determining individual-based follow-up periods. Very few patients with pT1aN0M0 RCC have tumor necrosis in RCC specimens, which was demonstrated in the present study (7/133 patients) and a previous study (8/293 patients) (6). However, if tumor necrosis is detected, the patients should be followed more closely than patients without tumor necrosis.

The present study has several limitations. First, this is a non-randomized, retrospective, single-center study. Therefore, a prospective study including a large number of patients is required to confirm these observations. However, the current study revealed an important finding; tumor necrosis was an independent predictor for recurrence in pT1aN0M0 RCC.

Histological tumor necrosis was the only independent predictor for recurrence in patients with pT1aN0M0 RCC. The frequency of tumor necrosis was low in patients with pT1aN0M0 RCC. However, patients with tumor necrosis in RCC specimens had a significantly higher risk for recurrence compared with those without tumor necrosis. Therefore, the presence of tumor necrosis may reflect an aggressive biological activity and be an effective predictor for recurrence in small RCCs.

## Figures and Tables

**Table I:** Comparison of clinicopathological factors between patients with recurrence and those without.

Variables	Patients with rec. (n=5)	Patients without rec. (n=128)	P-value
Age (years)	61±12	67±8	0.2636
Gender (male/female)	3.2	91/37	0.5930
Side (right/left)	4/1	63/65	0.1769
Tumor size (cm)	3.5±0.4	2.8±0.7	0.0390
ECOG PS (0 vs. ≤1)	3/2	112/16	0.0778
Subtypes of RCC (clear cell vs. others)	5/0	112/6	0.6203
Grade 3 component (+ vs. −)	1/4	12/116	0.4325
MVI (+ vs. −)	2/3	18/110	0.1114
Tumor necrosis (+ vs. −)	3/2	4/124	<0.0001
CRP (>0.3 vs. ≤0.3)	2/3	14/113	0.0515

Rec., recurrence; ECOG PS, Eastern Cooperative Oncology Group performance status; RCC, renal cell carcinoma; MVI, microvascular invasion; CRP, C-reactive protein.

**Table II:** Multivariate analysis for predicting recurrence in patients with pT1aN0M0 RCC (n=133).

	Univariate	Multivariate		
		
Variables	P-value	P-value	Odds ratio	Relative risk ratio 95% CI
Age	0.1591			
Gender	0.7189			
ECOG PS	0.1151			
Tumor side	0.2449			
Tumor size	0.0379	0.3622	2.355^a^	0.0373–14.866
Grade 3 component (+)	0.1353			
MVI (+)	0.0975			
Tumor necrosis (+)	0.0003	0.0143	14.286	1.701–125
CRP (≥0.3 mg/dl)	0.1061			

a By a 1 cm increase.

RCC, renal cell carcinoma; CI, confidence interval; ECOG PS, Eastern Cooperative Oncology Group performance status; MVI, microvascular invasion; CRP, C-reactive protein.

**Table III:** Comparison of clinicopathological factors between pT1bN0M0 (≤5 cm) patients with recurrence and those without.

Variables	Patients with pT1b tumor (≤5 cm) (rec.+) (n=8)	Patients with pT1b tumor (≤5 cm) (rec. −) (n=40)	P-value
Age (years)	65±10	60±13	0.2509
Gender (male/female)	8/0	25/15	0.0367
Side (right/left)	3/5	21/19	0.4386
Tumor size (cm)	4.6±0.3	4.4±0.3	0.0563
ECOG PS (0 vs. 1)	0/8	6/33	0.2349
Grade 3 (+ vs. −)	3/5	14/26	0.8926
MVI (+ vs. −)	5/3	16/24	0.2416
Tumor necrosis (+ vs. −)	4/4	6/34	0.0261
CRP (0.3> vs. 0.3)	5/3	11/29	0.0552

ECOG PS, Eastern Cooperative Oncology Group performance status; MVI, microvascular invasion; CRP, C-reactive protein.
